# Hypnotism in Russsia

**Published:** 1893-09

**Authors:** 


HYPNOTISM IN RUSSIA.—An official notice has been issued in Russia that "physicians shall have the right to make use of hypnotism in the treatment of their patients. In every case of the application they must inform the administrative authorities, at the same time giving the names of the physicians in whose presence the patient was hypnotized."

